# Integrative Multi-Omics Analysis Reveals the Genetic Architecture Landscape of the Blood Metabolome in Jersey Cattle

**DOI:** 10.3390/ani16152334

**Published:** 2026-07-31

**Authors:** Xinyi Zhang, Jun Teng, Zhujun Chen, Qin Zhang

**Affiliations:** Shandong Provincial Key Laboratory of Animal Biotechnology and Disease Control and Prevention, College of Animal Science and Veterinary Medicine, Shandong Agricultural University, Tai’an 271018, China; gd916@163.com (X.Z.);

**Keywords:** multi-omics integration, Jersey cattle, blood metabolome, cis-eQTL, mediation analysis

## Abstract

Metabolites are important intermediate molecular phenotypes that reflect physiological and biochemical processes within an organism. However, the molecular mechanisms linking genetic variation to metabolite abundance remain poorly understood in dairy cattle. Here, we integrated whole-genome sequencing, whole-blood transcriptomic, and plasma metabolomic data from 80 Jersey cattle to investigate the genetic regulation of circulating metabolites. After quality control, 10,696,212 high-quality SNPs, 15,559 expressed genes, and 841 stable plasma metabolites were retained for downstream analyses. Cis-eQTL mapping identified 1863 eGenes regulated by 1,340,105 significant cis-eQTLs. Transcriptome–metabolome association analysis further detected 258 significant gene–metabolite associations involving 148 genes and 177 metabolites. By integrating cis-eQTLs with gene–metabolite associations, mediation analysis identified 218 significant SNP–gene–metabolite trios involving 124 genes and 157 metabolites. Network analysis further identified several highly connected mediator genes, including *MEGF9*, *S1PR5*, and *CD27* and revealed two distinct mediation patterns, complete and partial mediation. Together, these findings indicate that gene expression serves as an important intermediate layer connecting genetic variation with circulating metabolites. This study provides a comprehensive multi-omics resource for investigating the genetic regulation of the blood metabolome in Jersey cattle and offers new insights into the molecular basis of metabolic variation.

## Simple Summary

Metabolites are small molecules in the blood that reflect many important biological processes, including energy use, nutrient metabolism, and health status. Understanding why the levels of these molecules differ among animals can help improve our knowledge of cattle biology and support future breeding programs. In this study, we integrated genomic, gene expression, and blood metabolite data from Jersey cattle to better understand how genetic variation influences metabolite levels. We found that many genetic variants affect metabolite levels indirectly through changes in gene expression. Some genetic effects were transmitted mainly through changes in gene expression, whereas others appeared to involve additional biological processes beyond the measured genes. The results provide new insight into how genetic variation influences metabolite abundance in Jersey cattle and improve our understanding of the biological connections between genes and metabolites. These findings also provide a valuable resource for future studies investigating the molecular regulation of blood metabolites in Jersey cattle.

## 1. Introduction

Jersey cattle are an important dairy breed that is widely recognized for their distinct physiological and metabolic characteristics. Compared to many commercial dairy breeds, Jersey cattle exhibit differences in nutrient utilization, energy metabolism, and milk composition, making them an attractive population for investigating the biological basis of metabolic variation in dairy cattle [1]. Because lactation is accompanied by extensive changes in nutrient partitioning, lipid metabolism, amino acid utilization, and immune-related metabolic processes, circulating metabolites can provide informative intermediate phenotypes for characterizing systemic metabolic variation in dairy cattle [2]. Therefore, dissecting the genetic regulation of circulating metabolites in Jersey cattle may help identify candidate regulatory genes and metabolic pathways that could enhance our understanding of lactation biology and metabolome-mediated genetic regulation of economically relevant traits in Jersey cattle.

Circulating metabolites have emerged as important intermediate molecular phenotypes because they directly reflect physiological and biochemical processes occurring within an organism [3,4]. Recent metabolite genome-wide association studies (mGWAS) have identified numerous genetic loci associated with variation in metabolite abundance in both humans and livestock species [5,6]. However, translating these associations into specific candidate genes and biological mechanisms remains challenging, as most associated variants are located in non-coding regions and may exert their effects through complex regulatory processes. Consequently, additional molecular layers are required to bridge the gap between genetic variation and downstream metabolic phenotypes. Gene expression represents a key intermediate molecular phenotype linking genetic variants to downstream biological processes [7,8]. Expression quantitative trait locus (eQTL) mapping links genetic variants to changes in gene expression, providing an effective approach for identifying regulatory genes underlying genetic associations [9,10]. In recent years, multi-omics studies in dairy cattle and other farm animals have been increasingly conducted to investigate complex traits [9,11,12]. However, most previous studies have focused on pairwise omics integrations, such as genomic–transcriptomic or genomic–metabolomic analyses, whereas direct integration of genetic variation, gene expression, and metabolite abundance remains limited, particularly in Jersey cattle. Incorporating metabolomics can help link genetic variations and gene expressions to metabolic changes and provide a useful way to investigate the metabolome-mediated genetic regulations of economically relevant traits.

Among the tissues available for multiple omics profiling, whole blood is particularly suitable for integrative analyses because it serves as a major transport medium for nutrients, metabolites, hormones, and immune signals throughout the body [13,14]. In dairy cattle, blood metabolomic profiles can reflect systemic physiological processes related to energy balance, lipid transport, amino acid metabolism, immune status, and lactation-associated metabolic adaptation [15]. These features make blood a useful and accessible tissue for investigating the regulatory basis of circulating metabolic variation. Thus, whole blood provides a practical blood-based molecular layer for linking genetic variations, gene expressions, and circulating metabolite abundance in the same individuals. Another important advantage of using blood for multi-omics study is that it can be easily sampled in live animals with very low cost and therefore suitable for large scale studies. 

**Figure 1:**
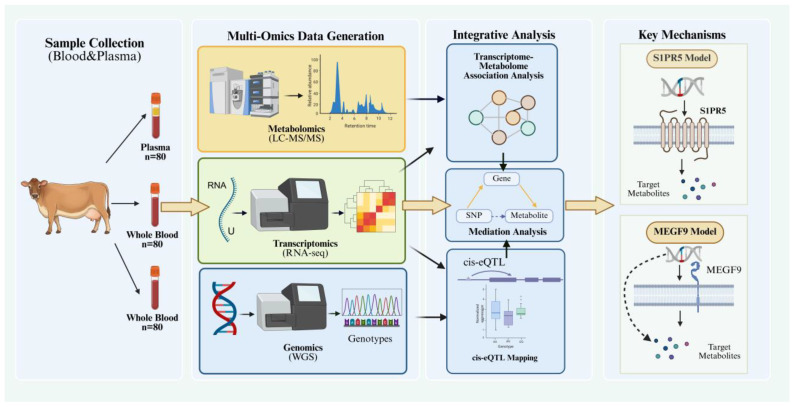
Study workflow and multi-omics data summary. Schematic overview of the integrative analysis of WGS, whole-blood RNA-seq, and plasma LC-MS/MS metabolomics data from 80 Jersey cattle. The workflow includes cis-eQTL mapping, gene–metabolite association analysis, and mediation analysis to identify candidate SNP–gene–metabolite regulatory trios. Created in BioRender. Zhang, X. (2026) https://BioRender.com/12c4qgc (accessed on 20 July 2026).

The central hypothesis of this study was that part of the genetic regulations of circulating metabolite abundances in Jersey cattle is mediated through changes in gene expressions in whole-blood. To test this hypothesis, genomic, whole-blood transcriptomic, and plasma metabolomic data from 80 Jersey cattle were collected and integrated using the analytical framework summarized in Figure 1. Specifically, the analysis aimed to identify cis-regulatory variants affecting gene expressions in blood, characterize expression–metabolite associations, and prioritize SNP–gene–metabolite regulatory trios through mediation analysis. These results provide a multi-omics resource for understanding the genetic architecture of the blood metabolome and establish a foundation for future investigations of the metabolome-mediated genetic regulations of economically relevant traits in Jersey cattle.

## 2. Materials and Methods

### 2.1. Animals and Sample Collection

A cohort of 80 Jersey dairy cows from a single commercial herd (Jinan Jiabao Dairy Co., Ltd., Jinan, China) was used in this study. All animals were housed in the same pen and in the first parity with ages ranging from 22 to 24 months. All biological samples were collected on the same day after the first morning milking but before the first morning feeding. On the sampling day, the cows were at approximately the same milking stage with days in milk ranging from 120 to 150 days. For genomic DNA (gDNA) and RNA-seq analysis, approximately 10 mL of whole blood was drawn from the tail vein of each cow into a vacutainer tube containing EDTA. These samples were immediately flash-frozen on dry ice. For non-targeted metabolomic analysis, a separate 10 mL blood sample was collected from the tail vein into a vacutainer tube containing EDTA as an anticoagulant. These samples were immediately processed by centrifugation at 3000 *g* for 10 min at 4 °C, and then 400 µL of the plasma supernatant was transferred to a new cryotube and immediately flash-frozen in liquid nitrogen for 15 min. All collected biological samples were subsequently stored at −80 °C until further processing.

### 2.2. RNA Sequencing and Expression Profiling

Total RNA was isolated from the 80 whole blood samples without prior cellular separation using TRNzol reagent (TIANGEN, Beijing, China) following the manufacturer’s protocol. Transcriptome libraries were constructed and sequenced on an Illumina NovaSeq 6000 platform to generate paired-end 150bp reads. Raw sequencing reads were processed using fastp (v0.23.2) [16] to remove adapter sequences and low-quality data. Specifically, we applied polyG tail trimming (-g) and filtered reads with a base quality score < 5 (-q 5), unqualified bases limit > 50% (-u 50), N base limit > 15 (-n 15), and minimum read length < 150 bp (-l 150) to retain high-quality reads with the expected full read length. Clean reads were aligned to the bovine reference genome (ARS-UCD1.2) using the HISAT2 (v2.2.1) [17] with default parameters. The output BAM files were sorted using SAMtools (v1.15.1) [18]. All 80 samples passed quality control with mapping rates > 80%. Gene expression levels were quantified using StringTie (v2.1.4) [19] in expression estimation mode (-e -B). Expression values were calculated as Transcripts Per Million (TPM). We retained expressed genes, defined as having TPM ≥ 0.1 in at least 20% of the samples according to Cattle GTEx guidelines [9] for downstream eQTL mapping.

### 2.3. Low-Coverage WGS and Genotype Imputation

Genomic DNA (gDNA) was extracted from the 80 whole blood samples using the GenoPrep Blood DNA Rapid Extraction Kit (MolBreeding Biotech, Shijiazhuang, China) following the manufacturer’s protocol. The samples were sequenced via low-coverage whole-genome sequencing (lcWGS) on an MGISEQ T7 platform (MGI). Raw sequencing reads were processed using fastp (v0.23.2) [16] to remove adapter sequences and filter low-quality reads. The filtering parameters were set to N base limit < 10 (-n 10), base quality score < 20 (-q 20), and unqualified bases limit > 40% (-u 40). Clean reads were aligned to the bovine reference genome (ARS-UCD1.2) using the mem algorithm in BWA (v0.7.17) [20] with read groups assigned. The BAM files were sorted, and PCR duplicates were removed using SAMtools (v1.15.1) [18]. Genotype imputation was performed using GLIMPSE2 [21], following a previously established pipeline [22]. The reference panel consisted of 210 Jersey cattle with WGS data from the 1000 Bull Genomes Project (Run 8) [23]. The imputation pipeline included target region chunking (GLIMPSE2_chunk), phasing (GLIMPSE2_phase), and ligating (GLIMPSE2_ligate). Imputed VCF files from all chromosomes were concatenated using bcftools. Post-imputation quality control was conducted using PLINK (v1.9) [24]. We retained strict biallelic single-nucleotide polymorphisms (SNPs) and filtered variants based on the following thresholds: minor allele frequency (MAF) > 0.05 and Hardy–Weinberg equilibrium (HWE) *p*-value > 1 × 10^−6^.

To systematically evaluate the reliability of genotype imputation from low-coverage sequencing data, we performed a downsampling-based validation analysis. Briefly, 11 publicly available Jersey cattle with high-coverage whole-genome sequencing data (>15×; Table S1) were downloaded and used as a validation dataset, with the original high-confidence genotypes treated as the ground-truth reference. BAM files from these individuals were randomly downsampled to approximately 1× sequencing depth using the DownsampleSam module implemented in Picard tools (v3.4.0; http://broadinstitute.github.io/picard/ (accessed on 20 July 2026)), thereby simulating the sequencing depth of the study population. Five chromosomes, BTA1, BTA5, BTA10, BTA15, and BTA20, with varying lengths (71.97–158.53 Mb) and variant densities (4011.67–4599.85 SNPs/Mb) were subsequently selected for genotype imputation using the same GLIMPSE2 pipeline described above. Imputation accuracy was evaluated by calculating Pearson correlation coefficients (r) between imputed genotype dosages and the corresponding true genotypes derived from the original high-coverage sequencing data. To further assess imputation performance across different allele frequency spectra, variants were grouped into minor allele frequency (MAF) bins, and the mean imputation accuracy within each interval was calculated separately.

### 2.4. Non-Targeted Metabolomic Profiling and Data Processing

Metabolites were extracted from the 400 µL plasma aliquots using 700 µL of a pre-chilled solvent (methanol:acetonitrile:water, 4:2:1 *v*/*v*/*v*). The mixture was vortexed, incubated at −20 °C for 2 h, and centrifuged (25,000 *g*, 15 min, 4 °C). The supernatant was collected, re-solubilized, centrifuged again, and the final supernatant was transferred for analysis. A pooled quality control (QC) sample, created by mixing equal aliquots from all 80 samples, was injected every 10 samples to monitor instrument stability.

Metabolomic profiling was performed using a UPLC I-Class Plus system (Waters) coupled to a Q Exactive (QE) high-resolution mass spectrometer (Thermo Fisher Scientific, Waltham, MA, USA) with a BEH C18 column (1.7 μm, 2.1 × 100 mm). Data was acquired in both positive (ESI+) and negative (ESI-) ion modes. Raw data files were processed using Compound Discoverer 3.3 (Thermo Fisher Scientific) for peak picking, alignment, and metabolite identification against the BMDB, mzCloud, and ChemSpider databases (precursor mass tolerance < 5 ppm). The resulting feature matrix was processed using the metaX tool (v1.4.2), which included: (1) Probabilistic Quotient Normalization (PQN) [25]; (2) Quality Control-Based Robust LOESS Signal Correction (QC-RLSC) for batch effect removal [26]; and (3) filtering to remove features with a coefficient of variation (CV) > 30% in the pooled QC samples. For downstream association analysis, the final metabolite abundances were log-transformed, standardized (scaled to a mean of 0 and SD of 1), and corrected for technical covariates (e.g., analytical batch) using a linear model. The resulting residuals were used for the subsequent analyses. Post-imputation quality control restricted the dataset to autosomal variants with a missing rate of 0, MAF ≥ 0.05, and HWE *p* ≥ 1.0 × 10^−6^, yielding a final dataset of 10,696,212 variants for subsequent association testing.

### 2.5. cis-eQTL Mapping and Analysis

Prior to eQTL mapping, expressed genes were filtered to retain those with transcripts per million (TPM) > 0.1 in at least 20% of the samples. The expression matrix was quantile-normalized using the preprocessCore package in R [27] and subjected to rank-based inverse normal transformation (INT) across samples. To account for hidden technical and biological confounding factors, we calculated Probabilistic Estimation of Expression Residuals (PEER) factors using peertool [28]. We computed an initial set of 30 factors and selected the top three as covariates based on the elbow method observed in the factor relevance plot (Figure S1). cis-eQTL mapping was performed on the 80 whole blood samples using a univariate linear mixed model (--uvlmm) implemented in GMAT (https://github.com/chaoning/GMAT (accessed on 20 July 2026)). The model incorporated the top three PEER factors as covariates and the genomic relationship matrix (GRM) to control for population structure. The analysis tested for associations between SNP genotypes and gene expression, defining cis-variants as those located within a ±1 Mb window of a gene’s transcription start site (TSS). To control the false discovery rate (FDR), *p*-values were adjusted using the Benjamini–Hochberg (BH) method [29]. Associations with an FDR < 0.05 were considered significant cis-eQTLs, and genes associated with at least one significant SNP were defined as eGenes. To identify independent cis-eQTLs for each eGene, we conducted a conditional and joint (COJO) analysis using GCTA-COJO [30]. The conditional selection (--cojo-slct) utilized all SNPs within the 1 Mb cis-window of the eGene that had an initial association *p*-value < 1 × 10^−5^. The functional consequences of the identified independent cis-eQTLs were annotated using the Ensembl Variant Effect Predictor (VEP) [31] to determine their genomic context.

### 2.6. Allele-Specific Expression Analysis

Allele-specific expression (ASE) analysis was performed to assess allelic imbalance in gene expression and provide complementary evidence for cis-regulatory activity. ASE was quantified using GATK ASEReadCounter (v4.0.8.1). Heterozygous SNPs present in at least five samples and supported by sufficient read depth (≥10 reads per allele and ≥2% of total reads supporting the minor allele) were tested for allelic imbalance using a binomial test with Benjamini–Hochberg FDR correction. SNPs with FDR < 0.05 were considered significant ASE loci, and allelic fold change (aFC) was estimated using phASER (v1.1.1) [32].

### 2.7. Transcriptome–Metabolome Association Analysis

To test for associations between gene expression and plasma metabolite levels, we restricted our analysis to the identified cis-eGenes to reduce the multiple testing burden. The association testing was conducted using a univariate linear mixed model (--uvlmm) implemented in GMAT2. In this model, the normalized abundance of each metabolite was treated as the phenotypic trait, and the normalized expression level of the cis-eGene was included as a fixed effect. To control for population structure and genetic relatedness, the genomic relationship matrix (GRM) was included as a random polygenic effect. *p*-values were adjusted using the Benjamini–Hochberg procedure, and associations with a false discovery rate (FDR) < 0.05 were considered statistically significant. Genes significantly associated with metabolite levels were then subjected to Gene Ontology (GO) and Kyoto Encyclopedia of Genes and Genomes (KEGG) enrichment analysis using the Database for Annotation, Visualization and Integrated Discovery (DAVID) [33]. Bos taurus was set as the background species. Terms and pathways with a raw *p*-value < 0.05 were considered significantly enriched.

### 2.8. Mediation Analysis

To evaluate whether gene expression mediates the association between cis-eQTLs and metabolite abundances, we performed a mediation analysis on identified regulatory trios. Each trio consisted of a cis-eQTL, its target eGene, and the associated metabolite. For each trio, two linear regression models were constructed: one modeling the effect of the cis-eQTL on gene expression (the mediator), and another modeling the joint effect of the cis-eQTL and gene expression on metabolite abundance (the outcome). The mediation analysis was conducted using the R Mediation Package (v4.5.0) [34]. The Average Causal Mediation Effect (ACME) and Average Direct Effect (ADE) were estimated. The statistical significance of the ACME was tested using a non-parametric bootstrap method with 10,000 resamples. To control for multiple testing across all evaluated trios, the raw ACME *p*-values were adjusted using the Benjamini–Hochberg (BH) method. A mediation effect was considered statistically significant at a false discovery rate (FDR) < 0.05.

## 3. Results

### 3.1. Summary of Multi-Omics Datasets and Quality Control

We first evaluated the quality and scale of the multi-omics data generated for the 80 Jersey cows. The RNA-seq analysis of whole blood samples yielded a total of 3.9 billion clean reads, with an average mapping rate of 93.35% to the bovine reference genome (Table S2). After applying expression filters (TPM ≥ 0.1 in at least 20% of the samples), we identified 15,559 expressed genes that were retained for downstream analysis. 

**Figure 2:**
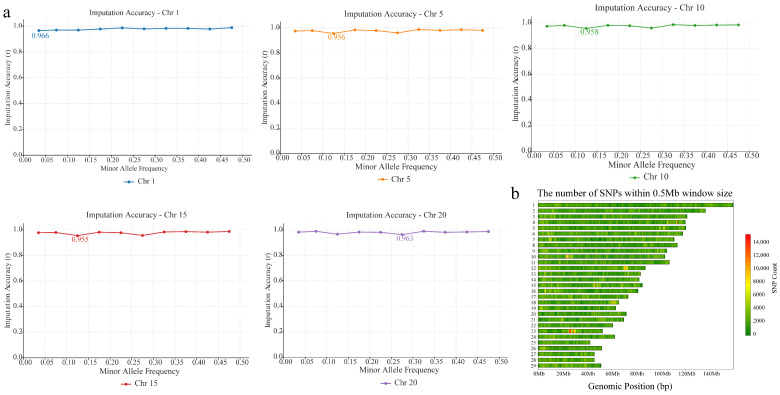
Genotype imputation performance and genome-wide SNP distribution. (**a**) Imputation accuracy across different minor allele frequency (MAF) bins for five representative chromosomes (BTA1, BTA5, BTA10, BTA15, and BTA20). The *y*-axis indicates imputation accuracy measured as the correlation (r) between imputed and observed genotypes. Numbers within each panel indicate the average imputation accuracy for the corresponding chromosome. (**b**) Distribution of SNP density across the bovine genome. SNP counts were calculated in consecutive 0.5 Mb genomic windows and are shown for all autosomes. Color intensity represents the number of SNPs within each window.

For the genomic data, low-coverage WGS followed by imputation resulted in a high-density genotype dataset. A downsampling-based validation analysis using 11 independent Jersey cattle with high-coverage whole-genome sequencing data demonstrated high imputation accuracy, with an average genotype dosage correlation of 0.977 between imputed and true genotypes across the five evaluated chromosomes. As expected, imputation accuracy increased with minor allele frequency and remained high for common variants (Figure 2a). After strict quality control filtering (MAF > 0.05; HWE *p* > 1 × 10^−6^), a total of 10,696,212 high-quality SNPs were retained. The chromosomal distribution and density of these variants (0.5 Mb window size) are visualized in Figure 2b, confirming uniform coverage across the genome. Finally, the non-targeted metabolomic profiling of plasma samples identified 841 stable metabolites after quality control and filtration. These high-quality multi-omics datasets were then utilized for the subsequent association studies.

### 3.2. Identification and Annotation of cis-eQTLs

**Figure 3:**
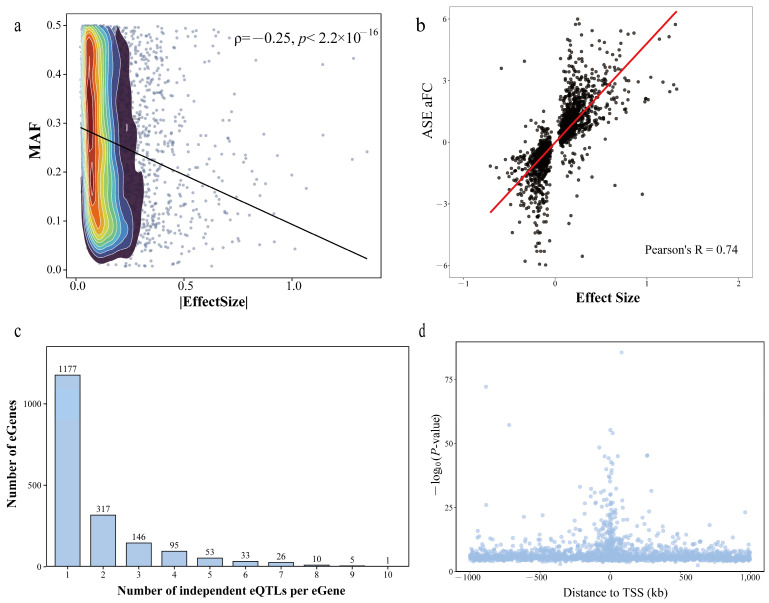
Characteristics and validation of cis-eQTLs identified in Jersey cattle blood. (**a**) Relationship between minor allele frequency (MAF) and absolute cis-eQTL effect size. The black line represents the fitted regression, and the Spearman correlation coefficient (ρ) is shown. (**b**) Concordance between cis-eQTL effect sizes and allele-specific expression (ASE)-based allelic fold changes (aFC). Each point represents a cis-eQTL–gene pair, and the red line indicates the fitted linear regression. (**c**) Distribution of the number of independent cis-eQTLs detected per gene. Numbers above bars indicate the corresponding gene counts. (**d**) Genomic distribution of independent cis-eQTLs relative to transcription start sites (TSSs). Each point represents an independent cis-eQTL, with the *x*-axis indicating distance from the TSS and the *y*-axis showing the significance level of association.

We performed cis-eQTL mapping to identify genetic variants regulating gene expressions in whole blood. This analysis identified 1863 eGenes regulated by 1,340,105 significant cis-eQTLs (FDR < 0.05). These eGenes account for 11.97% of all expressed autosomal genes in the dataset. The absolute effect sizes of lead variants were significantly and negatively correlated with minor allele frequencies (Figure 3a), consistent with the selection-driven relationship between effect size and allele frequency. To further validate these signals at the individual level, we performed allele-specific expression (ASE) analysis. The calculated allelic fold changes (aFC) from ASE data showed a high correlation with our mapping effect sizes (Figure 3b), confirming the technical robustness of the identified cis-eQTLs. To distinguish independent regulatory effects from those caused by linkage disequilibrium, we conducted a conditional and joint analysis using GCTA-COJO (Figure 3c). This revealed 3409 independent cis-eQTLs across 1863 eGenes. Among these, 1177 eGenes were associated with a single independent cis-eQTL, while 317, 146, and 223 eGenes were associated with two, three, and four (or more) independent cis-eQTLs, respectively. Spatially, these independent cis-eQTLs were highly concentrated near the Transcription Start Sites (TSS), with smaller *p*-values observed for variants located closer to the TSS (Figure 3d). Furthermore, functional annotation of the independent cis-eQTLs using VEP illustrated their genomic distribution (Table S3). Most of them were located in intergenic regions (*n* = 1916) or intronic regions (*n* = 1145), which collectively accounted for approximately 89% of the annotated signals. The rest were in regulatory regions, including upstream gene variants (*n* = 124) and downstream gene variants (*n* = 124). Direct coding or transcript-modifying variants were less frequent but included specific functional categories such as missense variants (*n* = 30) and UTR variants (*n* = 26; including 5′ and 3′ UTRs). This distribution suggests that most independent cis-eQTL may affect gene expressions through non-coding mechanisms, such as nearby regulatory elements, intronic regulatory activity, or potential effects on RNA splicing and transcript processing, rather than through direct changes in protein-coding sequences.

### 3.3. Associations Between Transcriptome and Metabolome

**Figure 4:**
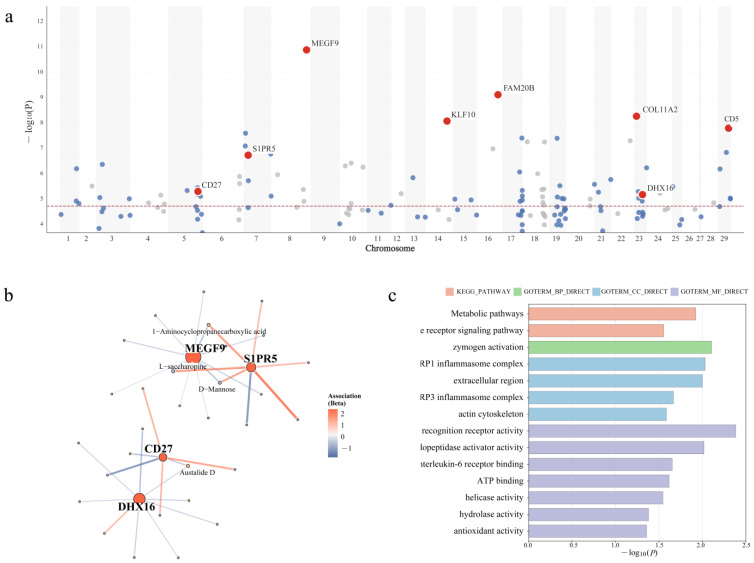
Transcriptome–metabolome associations in Jersey cattle blood. (**a**) Genome-wide distribution of genes significantly associated with plasma metabolites. Each point represents a gene–metabolite association, with the *y*-axis indicating −log10(*p*-value). The dashed line indicates the significance threshold, and the red labeled points highlight representative metabolite-associated genes. (**b**) Subnetwork of representative hub genes and their associated metabolites. Nodes represent genes and metabolites, and edges represent significant associations. Edge colors indicate the direction and magnitude of regression coefficients. (**c**) Functional enrichment analysis of genes significantly associated with metabolites. The enriched Gene Ontology (GO) and KEGG pathway terms are shown, with bar lengths representing enrichment significance.

To investigate the relationship between gene expression and circulating metabolite abundance, we performed transcriptome–metabolome association analysis focusing on the 1863 eGenes identified from the cis-eQTL mapping. Using a univariate linear mixed model (uvLMM), we identified 258 significant gene–metabolite associations (FDR < 0.05), involving 148 unique genes and 177 metabolites (Figure 4a; Table S4). These associations were distributed across multiple genomic regions and collectively revealed extensive connections between blood gene expression and the plasma metabolome in Jersey cattle. Notably, several genes exhibited associations with multiple metabolites, suggesting the presence of shared regulatory mechanisms linking transcriptional variation to diverse metabolic processes. 

To further characterize the architecture of these associations, we constructed a transcriptome–metabolome interaction network based on all significant gene–metabolite pairs (Figure 4b). Network analysis revealed substantial heterogeneity in gene connectivity, with a small number of highly connected genes accounting for a disproportionate number of associations. Among the annotated genes, *MEGF9*, *DHX16*, *S1PR5*, and *CD27* emerged as the major hub nodes, being associated with 12, 9, 7, and 6 metabolites, respectively. Examination of the metabolites connected to these hub genes revealed distinct association patterns. *MEGF9* was linked to metabolites involved in carbohydrate, amino acid, and lipid metabolism, whereas *S1PR5* showed preferential associations with several bioactive compounds and antioxidants. In contrast, *CD27* was primarily associated with metabolites related to energy metabolism, while *DHX16* displayed associations spanning multiple metabolite classes. These results suggest that a subset of genes may occupy central positions within the blood transcriptome–metabolome network and potentially coordinate variation across multiple metabolic processes.

To characterize the biological functions represented by these genes, we performed functional enrichment analysis. The results of the 148 metabolite-associated genes indicated significant enrichment of immune-related functions and pathways (Figure 4c, Table S5). The top enriched terms included pattern recognition receptor activity, NLRP1 and NLRP3 inflammasome complexes, and the NOD-like receptor signaling pathway. In addition, enrichments in the antioxidant activity and metabolic pathways were also observed. Together, these results provide exploratory functional clues suggesting that immune regulation and metabolic processes may be involved in blood metabolome variations in Jersey cattle.

### 3.4. Mediation Analysis Reveals Potential Regulatory Axes 

**Figure 5:**
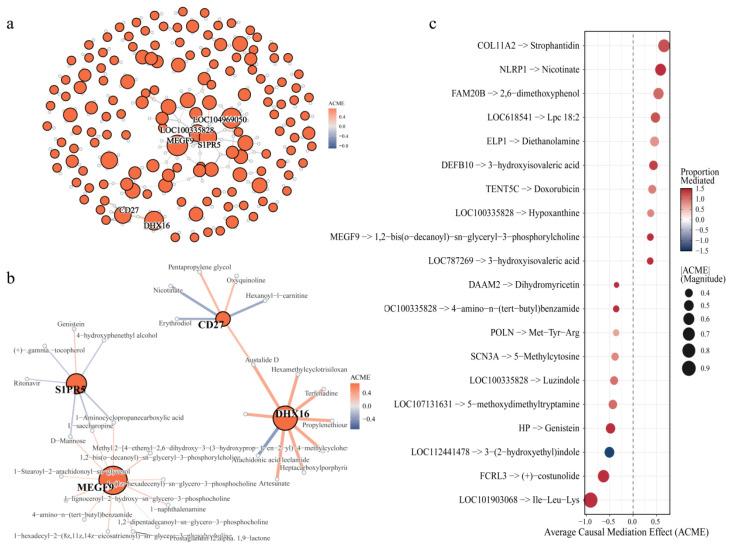
Mediation analysis of SNP–gene–metabolite relationships in Jersey cattle blood. (**a**) Global mediation network constructed from significant SNP–gene–metabolite trios. Nodes represent genes and metabolites, and edges represent significant mediation relationships. Edge colors indicate the direction and magnitude of the average causal mediation effect (ACME). (**b**) Subnetwork highlighting highly connected mediator genes, including *MEGF9*, *DHX16*, *S1PR5*, and *CD27*, together with their associated metabolites. Node size is proportional to network degree. (**c**) Representative SNP–gene–metabolite trios exhibiting the strongest positive and negative average causal mediation effects (ACME). Each dot represents one gene–metabolite mediation relationship, with dot color indicating the proportion mediated and dot size indicating the absolute ACME magnitude. The *x*-axis shows the ACME value.

To investigate whether the effects of cis-eQTLs on metabolite abundance were mediated through gene expression, we performed mediation analysis on 258 candidate SNP–gene–metabolite trios identified through the integration of cis-eQTL mapping and transcriptome–metabolome associations. After multiple-testing correction, 218 trios exhibited significant mediation effects (ACME FDR < 0.05), involving 124 unique genes and 157 metabolites (Table S6). Among the 218 significant trios, 208 (95.4%) showed full mediation pattern and 10 (4.6%) showed partial mediation pattern. These results indicate that full mediation represented the predominant regulatory pattern in this dataset. The mediation network constructed from these significant trios revealed widespread transcription-mediated genetic regulation of the plasma metabolome (Figure 5a). Within this network, *MEGF9*, *DHX16*, *S1PR5*, and *CD27* emerged as major hub genes, mediating the genetic regulation of 12, 9, 7, and 6 metabolites, respectively (Figure 5b), highlighting the different contribution of genes to metabolite regulation. The magnitude of mediation effects varied considerably across significant regulatory trios (Figure 5c). Among the strongest positive mediation effects were COL11A2–Strophantidin, NLRP1–Nicotinate, and FAM20B–2,6-dimethoxyphenol, whereas several trios exhibited strong negative mediation effects, including LOC101903068–Ile-Leu-Lys and FCRL3–(+)-costunolide.

**Figure 6:**
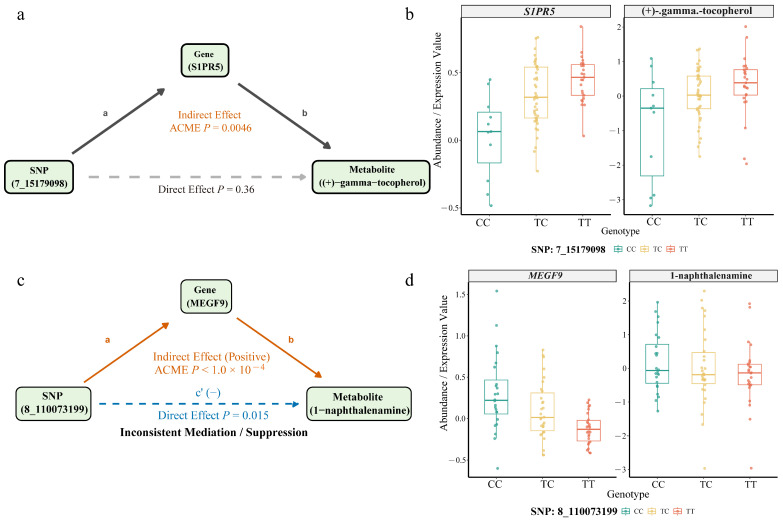
Representative full and partial mediation patterns identified in the blood multi-omics dataset. (**a**) Mediation model for the SNP (7_15179098)–S1PR5–(+)-gamma-tocopherol trio, illustrating a full mediation effect. Path coefficients and corresponding significance levels are indicated. (**b**) *S1PR5* expression and (+)-gamma-tocopherol abundance stratified by genotype at SNP 7_15179098. (**c**) Mediation model for the SNP (8_110073199)–MEGF9–1-naphthalenamine trio, illustrating a partial mediation effect in which both mediated and direct genetic effects contribute to metabolite variation. Path coefficients and corresponding significance levels are indicated. (**d**) *MEGF9* expression and 1-naphthalenamine abundance stratified by genotype at SNP 8_110073199. Boxplots show the median, interquartile range, and whiskers extending to 1.5 × the interquartile range. Solid arrows indicate the mediation paths (paths a and b), whereas dashed arrows indicate the direct effect of the SNP on the metabolite.

To further illustrate these mediation patterns, we selected representative regulatory trios for detailed examination. For the S1PR5–(+)–gamma–tocopherol axis, the indirect effect was significant whereas the direct effect was not, indicating that the genetic influence on metabolite abundance was primarily transmitted through changes in *S1PR5* expression (Figure 6a,b). In contrast, the MEGF9–1-naphthalenamine axis showed significant indirect and direct effects in opposite directions. As a result, the indirect effect mediated through *MEGF9* expression was largely offset by the direct genetic effect, producing an inconsistent mediation pattern (Figure 6c,d). These examples illustrate that genetic variants can influence metabolite abundance through both fully mediated and more complex regulatory relationships.

## 4. Discussion

Previous multi-omics studies in cattle and other farm animals have often focused on pairwise integrations, such as genomics and transcriptomics or genomics and metabolomics [5,35,36]. In this study, we established an integrated genomics, transcriptomics, and metabolomics framework in Jersey cattle blood and systematically characterized the relationships between genetic variations, gene expressions, and circulating metabolites. By combining cis-eQTL mapping, transcriptome–metabolome association analysis, and mediation analysis, we identified extensive transcriptionally mediated genetic effects on the plasma metabolome. This integrative design connects genetic variants, gene expressions, and metabolite abundances within a unified regulatory framework, providing a more direct way to interpret how genetic effects on circulating metabolites are mediated through transcriptional regulations.

The cis-eQTLs identified in Jersey cattle blood displayed several characteristic features that have been consistently observed in previous eQTL studies [11,12]. Variants with lower minor allele frequencies tended to exert larger effects on gene expression, supporting the expectation that strong regulatory variants are more likely to be constrained by purifying selection and therefore maintained at lower frequencies within populations [37,38]. The strong concordance between cis-eQTL effect sizes and ASE-derived allelic fold changes provided independent support for the authenticity of the detected regulatory signals. In addition, independent cis-eQTLs were strongly enriched near transcription start sites, with variants located closer to genes generally exhibiting stronger association signals. Consistent with this observation, functional annotation showed that most independent cis-eQTLs were located within intergenic and intronic regions rather than protein-coding sequences, indicating that regulatory variation predominantly acts through non-coding elements that influence transcriptional activity [7]. Similar enrichments of non-coding regulatory variants have been widely reported across human and livestock transcriptomic studies [8,9]. Collectively, these observations suggest that the genetic architecture of gene expression in Jersey cattle blood follows general regulatory principles observed across species. Despite the relatively modest sample size of 80 individuals, the recovery of these well-established eQTL features supports the robustness of the identified cis-regulatory signals and provides a reliable foundation for subsequent integrative analyses of gene expression and metabolite variation. However, because this study focused on cis-eGenes for downstream transcriptome–metabolome integration, trans-regulatory effects were not systematically evaluated. Given the larger multiple-testing burden and limited detection power of trans-eQTL mapping in a relatively small cohort, some trans-regulated genes or distal regulatory effects may have been missed.

Transcriptome–metabolome association analysis revealed widespread links between gene expression and circulating metabolites, suggesting close coordination between transcriptional activity and metabolic variation in blood [39]. The resulting network showed a clear hub structure, with a small number of genes connected to multiple metabolites, indicating that transcriptional regulation may influence groups of metabolic traits rather than isolated compounds. Among the hub genes, *MEGF9* encodes a multiple EGF-like domain-containing protein that is potentially involved in extracellular matrix organization, cell adhesion, and cell–matrix interactions [40]. Because extracellular matrix dynamics and cell–matrix signaling can influence membrane structure, lipid turnover, and intercellular communication [41], its association with multiple glycerophosphocholine-related metabolites may reflect a link between extracellular structural regulation and lipid-related metabolic variation in blood. *S1PR5* encodes a receptor for sphingosine-1-phosphate, a lipid mediator involved in immune-cell trafficking and vascular signaling [42]. Its associations with metabolites such as γ-tocopherol, genistein, and saccharopine may reflect a connection between immune-lipid signaling, antioxidant-related metabolism, and amino acid utilization. *CD27*, a receptor involved in lymphocyte activation [43], was associated with metabolites including nicotinate and hexanoyl-L-carnitine, suggesting that immune activation may be coupled with energy and cofactor-related metabolic variation. *DHX16*, an RNA helicase involved in RNA processing and innate immune signaling, may influence metabolite variation indirectly by affecting immune-related transcriptional or post-transcriptional programs [44]. Functional enrichment analysis further showed that these genes were mainly involved in immune signaling, receptor activity, extracellular processes, and metabolic pathways. These gene-specific patterns and enrichment results are closely aligned with the biological roles of blood. As a major immune compartment, blood contains diverse immune cell populations, while it also serves as the primary transport medium for circulating metabolites throughout the body [45]. Therefore, variation in blood gene expression may reflect not only local cellular processes but also broader physiological and metabolic states [46]. These findings suggest that gene expression represents an important intermediate layer linking cellular regulatory processes to systemic metabolic phenotypes and provide a basis for further investigating whether these relationships are genetically mediated.

Mediation analysis further indicated that a large proportion of genetic effects on circulating metabolites are transmitted through changes in gene expression, emphasizing the central role of transcriptional regulation in shaping the plasma metabolome. Similar observations have been reported in human multi-omics studies, where gene expression often serves as an intermediate molecular layer connecting regulatory variants to downstream complex traits [8,10]. These observations support a hierarchical regulatory model in which genetic variants influence metabolite abundance partly through their effects on gene expression. Notably, the mediation network exhibited a highly uneven structure, with most genes influencing only a few metabolites whereas a small number of mediators occupied central positions and were connected to multiple metabolites. *MEGF9*, the most highly connected mediator in the network, was involved in the genetic regulation of 12 metabolites. For some metabolites, such as 1-naphthalenamine, both significant mediation and direct genetic effects were detected, indicating a partial mediation pattern. Such results suggest that the measured transcript may capture only part of the underlying regulatory process and that additional mechanisms, including other molecular intermediates, distal regulatory effects, or post-transcriptional regulation, may also contribute to the observed genetic associations. Indeed, previous studies have shown that regulatory variants can influence complex traits through long-range chromatin interactions, trans-acting regulatory networks, or post-transcriptional processes that are not fully captured by local gene expression measurements [47,48,49]. In contrast, the regulatory relationships involving *S1PR5* were predominantly characterized by complete mediation. The effects of genetic variants on all seven associated metabolites were almost entirely transmitted through changes in *S1PR5* expression, with little evidence for residual direct genetic effects. Such patterns are consistent with the classical cis-regulatory model in which genetic variants primarily influence downstream phenotypes by modulating transcriptional activity through local regulatory elements, including promoters, enhancers, and chromatin-accessible regions [50,51]. Together, these observations highlight that the genetic regulation of circulating metabolites is not governed by a single mechanism. Some genetic effects appear to be transmitted primarily through changes in gene expression, whereas others likely involve additional regulatory processes that are not captured by the expression level of a single gene. Because circulating metabolites are closely related to energy balance, lipid metabolism, amino acid utilization, and immune status, these expression-mediated regulatory axes may provide useful clues for future studies of lactation physiology and economically relevant traits in dairy cattle. However, direct associations with production or health traits were not tested in the present study and should be evaluated in future studies using populations with matched phenotypic records.

Several limitations should be considered when interpreting these findings. First, although a substantial number of cis-eQTLs and mediation relationships were identified, the relatively modest sample size may have limited the detection of variants with small effects, including potential trans-regulatory effects, and reduced the power to resolve more complex regulatory architectures. Second, all analyses were performed using whole-blood transcriptomes. Because many circulating metabolites are influenced by metabolic processes occurring in other tissues, such as the liver, adipose tissue, and mammary gland, the regulatory mechanisms identified here likely represent only a subset of the pathways contributing to metabolite variation. Finally, mediation analysis provides statistical evidence for potential regulatory relationships but cannot by itself establish causal molecular mechanisms. Future studies incorporating transcriptomics data from more metabolically relevant tissues, such as the liver, mammary gland, and adipose tissue, would help distinguish blood-specific regulatory signals from systemic or tissue-specific metabolic regulation. Expanding the sample size is also necessary to improve the detection of small-effect regulatory variants and evaluate the reproducibility of the identified SNP–gene–metabolite axes. Moreover, functional experiments are also needed to validate the prioritized candidate genes and clarify their roles in metabolite regulation. Despite these limitations, the integrated multi-omics framework established here provides a useful resource for investigating the genetic regulation of metabolic traits in Jersey cattle and offers new insights into the molecular mechanisms linking genetic variation, gene expression, and circulating metabolites.

## 5. Conclusions

In conclusion, this study provides a comprehensive characterization of the regulatory relationships linking genetic variation, gene expression, and circulating metabolites in Jersey cattle blood. By integrating genomic, whole-blood transcriptomic, and plasma metabolomic data, we identified extensive cis-regulatory variation, widespread transcriptome–metabolome associations, and candidate SNP–gene–metabolite regulatory trios with significant mediation effects. These findings highlight gene expressions as an intermediate molecular layer that connects genetic variants with plasma metabolite abundances and explains partly how genetic effects on circulating metabolites are mediated through transcriptional regulations. At the same time, distinct mediation patterns suggest that genetic effects on metabolite abundance can arise through multiple regulatory mechanisms, including both expression-mediated and more complex regulatory processes. Overall, this study provides a blood-based multi-omics resource for Jersey cattle and offers candidate regulatory genes and pathways for future investigations of metabolic regulation and metabolome-mediated genetic regulation of complex traits in dairy cattle.

## Data Availability

The datasets generated and analyzed during the current study are not publicly available because they are part of an ongoing research project. Data may be made available from the corresponding author upon reasonable request and with permission from the project investigators.

## Supplementary Materials

The following supporting information can be downloaded at: https://www.mdpi.com/article/10.3390/ani16152334/s1, Table S1. Information of high-depth whole-genome sequencing samples used for genotype imputation accuracy validation. Table S2. Detailed sample-level statistics for RNA-sequencing data. Table S3. Functional annotation of independent cis-eQTLs. Table S4. Summary of significant associations between gene expression and plasma metabolites. Table S5. Functional annotation of metabolite-associated genes identified by transcriptome–metabolome association analysis. Table S6. Detailed results of mediation analysis for the identified regulatory trios. Figure S1. Factor relevance plot. A scree plot illustrating the percentage of variance explained by each computed factor. The elbow method, indicated by a marker, was used to select the top three factors as covariates for subsequent eQTL mapping.

## Abbreviations

ACME, average causal mediation effect ADE, average direct effect aFC, allelic fold change ASE, allele-specific expression ATAC-seq, assay for transposase-accessible chromatin with sequencing BH, Benjamini–Hochberg BMDB, bovine metabolome database COJO, conditional and joint analysis CV, coefficient of variation eGene, expression quantitative trait gene eQTL, expression quantitative trait loci GCTA, genome-wide complex trait analysis GLIMPSE, genotype likelihoods imputation and phasing method GO, Gene Ontology GRM, genomic relationship matrix KEGG, Kyoto Encyclopedia of Genes and Genomes lcWGS, low-coverage whole-genome sequencing LD, linkage disequilibrium mGWAS, metabolite genome-wide association study PEER, probabilistic estimation of expression residuals PQN, probabilistic quotient normalization QC, quality control QC-RLSC, quality control-based robust LOESS signal correction TPM, transcripts per million TSS, transcription start site uvLMM, univariate linear mixed model VEP, variant effect predictor WGS, whole-genome sequencing.
