# Supplementary material for: Integrative Multi-Omics Analysis Reveals the Genetic Architecture Landscape of the Blood Metabolome in Jersey Cattle
Source: Animals (Basel). 2026 Jul 31;16(15):2334. doi: 10.3390/ani16152334 (PMC13463560; doi:10.3390/ani16152334)

Figure. S1. Factor relevance plot. A scree plot illustrating the percentage of variance explained by each computed factor. The elbow method, indicated by a marker, was used to select the top three factors as covariates for subsequent eQTL mapping.

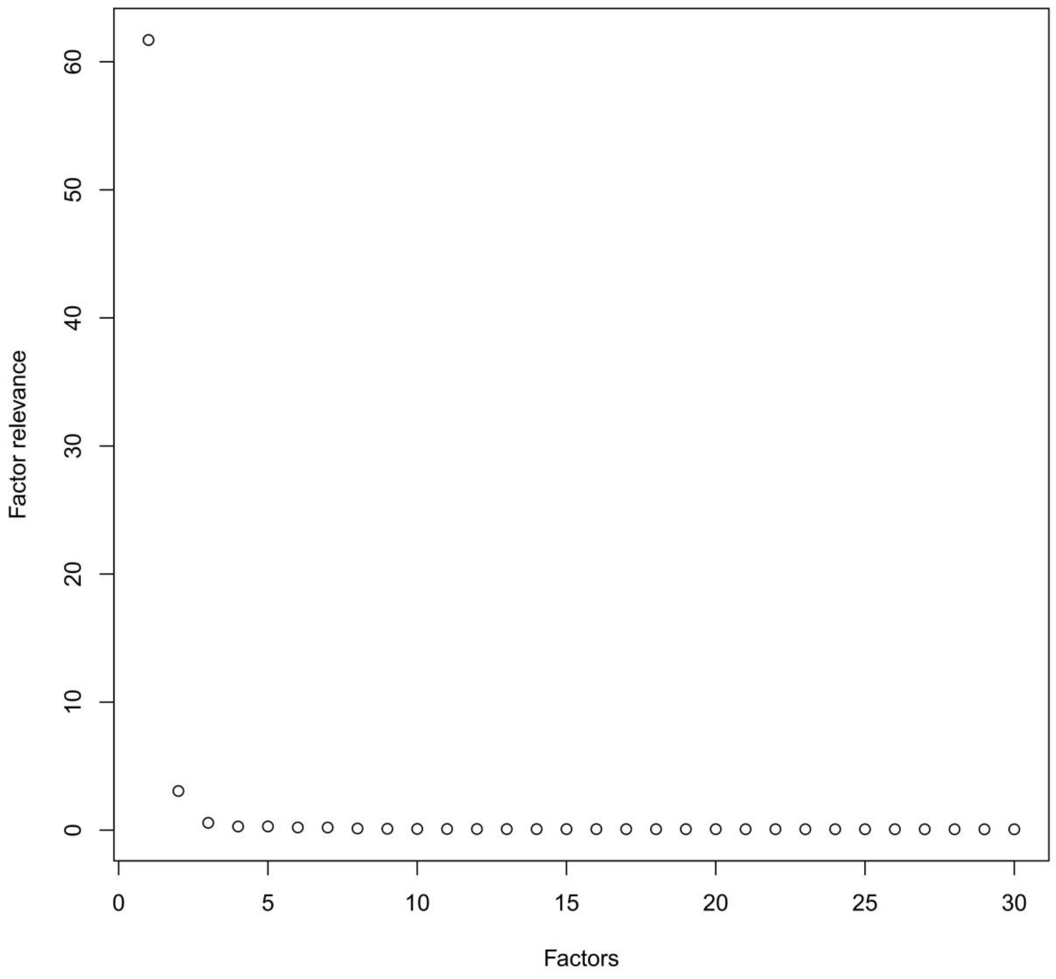

Supplement: Supplementary file 1 [file animals-16-02334-s001.zip › animals-4386279-supplementary.pdf]
